# Insights into gold nanoparticles as a mucoadhesive system

**DOI:** 10.1038/s41598-018-32699-2

**Published:** 2018-09-25

**Authors:** Mathieu Ouellette, Florence Masse, Mathilde Lefebvre-Demers, Quentin Maestracci, Philippe Grenier, Robert Millar, Nicolas Bertrand, Manuel Prieto, Élodie Boisselier

**Affiliations:** 1 CUO-Recherche, Hôpital du Saint-Sacrement, Centre de recherche du CHU de Québec and Département d’ophtalmologie, Faculté de médecine, Université Laval, Québec, Québec, G3K 1A3 Canada; 2 Centre de recherche du CHU de Québec and Faculté de pharmacie, Université Laval, Québec, Québec, G3K 1A3 Canada; 3 SRC Geoanalytical Laboratories, Saskatoon, Saskatchewan, S7N 2X8 Canada; 4 CQFM-IN and IBB-Institute for Bioengineering and Biosciences, Instituto Superior Técnico, Universidade de Lisboa, Lisboa, Portugal

## Abstract

A large number of drugs are administered on different mucosal surfaces. However, due to the poor mucoadhesion of the current formulations, their bioavailability is often very low. The development of efficient mucoadhesive drug delivery systems is thus crucial for improving the performance of these drugs. The mucoadhesive properties of gold nanoparticles were investigated. First, two types of gold nanoparticles were synthesized: AuNP1 and AuNP2. AuNP1 only contain internal thiol groups on their metallic core, and AuNP2 contain both internal and peripheral thiol groups. Different protocols based on an adapted quantitative colorimetric method, UV-visible and fluorescence spectroscopies were then developed to gather information on their mucoadhesive properties. Moreover, a global correction factor for the inner filter effect in spectrofluorimetry was proposed, and the data obtained were compared to those commonly used in the literature. Mucins deeply interact with AuNP1, perturbing their core, whereas they remain at the periphery of AuNP2. The quantitative method suggests that a larger number of mucins interact with AuNP2. The establishment of this protocol could be applied to assess the mucoadhesive properties of other stable molecules. This mucoadhesive property of gold nanoparticles could be combined with their drug delivery ability in order to improve the medication administered on mucosa.

## Introduction

Mucoadhesion is the ability to adhere to mucosal tissues, such as oral, buccal, nasal, vaginal and ocular mucosa (for reviews, see^1–5^). Mucoadhesive systems can be used to increase the retention time of drugs near the mucosa and thus improve their efficacy^6^. For example, 90% of the drugs used in ophthalmology are administered with ocular drops onto the mucosal layer of the cornea^7,8^. However, the bioavailability of such drugs is often low, due to poor mucoadhesion of the current formulations. For example, only 0.0006% to 0.02% of the active molecules administered onto the cornea are able to reach the anterior chamber of the eye, where most intended drug targets are located^9–12^. The field of buccal drug delivery also encounters difficulties with the mechanical effect of the tongue and the continuous dilution by saliva, leading to a low residence time of drugs^13,14^. The development of efficient mucoadhesive drug delivery systems is thus a crucial area to improve the bioavailability and therapeutic performance of these drugs. Mucins are the main family of proteins found in the mucosa and are strongly implicated in mucoadhesion due to two specific properties^15,16^. First, mucins are negatively charged and can interact electrostatically with positively-charged systems. Secondly, some regions of mucins are rich in cysteines (>10% of the amino acids) and therefore in thiol groups (-SH)^15^, leading to the formation of many disulfide bonds (S-S)^16^. Mucoadhesion can arise from the reorganization of these free thiol groups or intra-protein disulfide bonds^17^ with thiol groups on thiomers (polymers with thiol groups)^18^.

Interactions between thiol groups and mucins suggest that functionalized gold nanoparticles (AuNP) could be mucoadhesive due to the multiple thiol groups present on their metallic surface. Indeed, thiol groups stabilizing the gold core could interact with mucins and improve their mucoadhesive properties. Moreover, the thiol groups of mucins could also interact directly with the gold core to form gold-sulfur bonds (Au-S). AuNP are of utmost interest in nanomedicine (for reviews, see^19–26^). For example, drugs can be encapsulated in AuNP before being more efficiently released at their site of action. The vectorization of anticancer agents is one of many examples^27–29^. Different types of AuNP have also been developed to treat cancerous tumors by thermal phototherapy^30^. Similarly, silica-gold nanoparticles were also used in thermal phototherapy to successfully treat atherosclerosis in a clinical trial^31^. In imagery, new contrast agents such as gold nanospheres or nanorods were developed for X-ray imaging and computed tomography^32,33^. However, to the best of our knowledge, the mucoadhesion of AuNP has not been previously explored.

The mucoadhesive properties of AuNP were investigated in this paper. Two types of AuNP were synthesized; one with internal thiol groups near the metallic core and the other one with both internal and peripheral thiol groups. Different methodologies, including an optimised quantitative colorimetric method (PAS coloration), UV-visible and fluorescence spectroscopies were developed to assess their mucoadhesive properties. Moreover, a global formalism was described for the inner filter effect, allowing the correction of the re-absorption of the light by AuNP during fluorescence measurements.

## Results and Discussion

Two types of AuNP were synthesized and characterized. Their mucoadhesive properties were then studied by a quantitative colorimetric method, UV-visible and fluorescence spectroscopies.

### Synthesis of gold nanoparticles and ligand exchange

#### Synthesis of AuNP1

The Brust synthesis method is based on the use of thiolated ligands, allowing a rapid and strong stabilization of the gold core by thiol groups^34^. Ionic and covalent characters of the strong gold-sulfur bond (Au-S) lead to stable nanoparticles^35^. The AuNP here described were stabilized by thiolated polyethylene glycol (PEG) groups. PEG is a non-toxic ligand that is also used to provide water solubility to nanoparticles^36^. The AuNP synthesized using a modified Brust method do not require the use of TOAB, an ionic stabilizing/phase transfer agent, as their synthesis is performed in water where both gold salt (HAuCl_4_) and PEG are soluble. This procedure allows avoiding the use of cationic surfactants whose toxicity is due to their binding to the negatively charged components of cell membranes^37^.

**Figure 1:**
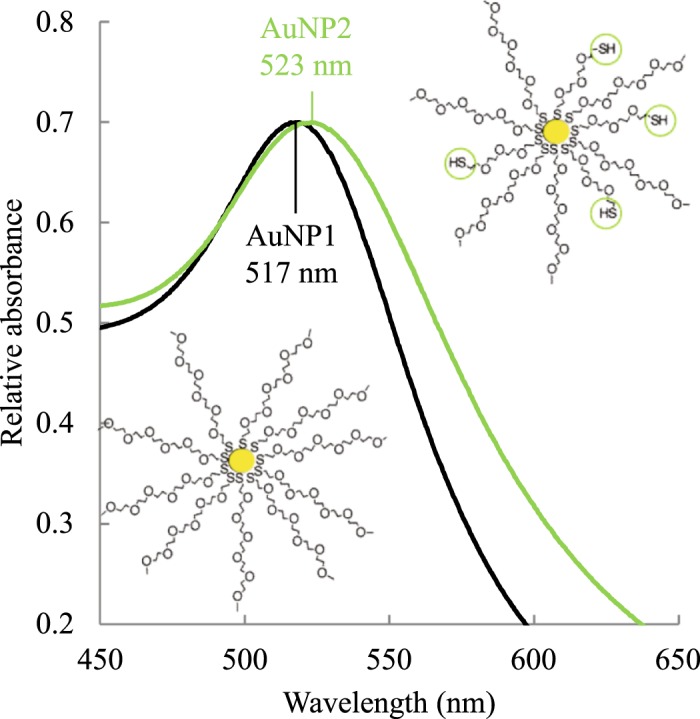
UV-visible spectra of AuNP1 (internal thiol groups, black spectrum) and AuNP2 (internal and peripheral thiol groups, green spectrum).

AuNP have a very characteristic absorbance due to the surface plasmon resonance phenomenon. Indeed, when the diameter of AuNP is much smaller than the wavelength of the light, a resonance of surface electrons occurs with the electromagnetic component of the light. The oscillation frequency of these electrons is in the visible region and a plasmon band is thus observable in UV-visible spectroscopy. AuNP1 show a UV-visible absorbance peak at 517 nm that is characteristic of their surface plasmon resonance (Fig. 1)^20^. These AuNP are soluble and stable in water, but also in different organic solvents such as dichloromethane or methanol.

#### Ligand exchange and synthesis of AuNP2

Despite the strength of the Au-S bond, it remains dynamic and allows the exchange with other thiol groups onto the metallic surface^38^. Indeed, in the presence of another thiol group, an exchange can occur onto the metallic surface. This concept was used to generate AuNP2. A ligand exchange was performed with PEG double thiol (details in the Methods section) to introduce thiol groups in the AuNP periphery. A PEG double thiol of 1000 g.mol^−1^ (half the size of PEG 2000) was chosen in order to minimize the risk of crosslinking and aggregation.

A bathochromic shift of the plasmon band was observed from 517 to 523 nm on the UV-visible spectrum of AuNP2 (Fig. 1 and Table 1). This shift indicates that the chemical environment around the gold core is modified due to the presence of PEG double thiol^39^.

**Table 1:** Characterization of AuNP1 and AuNP2.

	AuNP1	AuNP2
Plasmon band maximum	517 nm	523 nm
Metallic core diameter	7.2 ± 3.0 nm	7.8 ± 2.9 nm
Hydrodynamic diameter	36 ± 4 nm	35 ± 7 nm
Zeta potential	−13 ± 3 mV	0 ± 4 mV
% Au atoms	32.1%	30.5%
% S atoms	1.33%	1.83%
Number of internal thiol groups	2689	2689
Number of peripheral thiol groups	0	1205
Estimated molecular weight	7 460 000 g.mol^−1^	6 255 000 g.mol^−1^
Molar extinction coefficient	2.78 × 10^7^ L.mol^−1^.cm^−1^	1.57 × 10^7^ L.mol^−1^.cm^−1^

Data for metallic core diameters, hydrodynamic diameters and zeta potentials are reported as mean ± standard deviation.

The diameter of the metallic core of AuNP1 and AuNP2, determined by TEM (details in the Methods section), was respectively 7.2 ± 3.0 and 7.8 ± 2.9 nm (Supplementary Fig. S1 and Table 1). The mean diameter remained unchanged before and after the ligand exchange. This result was expected because the ligand exchange around the gold core does not lead to the destabilization or aggregation of AuNP^38^. The hydrodynamic diameter, determined by DLS (details in the Methods section), remained also comparable with 36 ± 4 and 35 ± 7 nm for AuNP1 and AuNP2, respectively (Table 1). The hydrodynamic diameter describes the diameter of the metallic core with the ligands and its solvation sphere. The introduction of PEG double thiol did not drastically change the size of AuNP2 and the diameter of AuNP1 and AuNP2 can be considered similar. Moreover, in accordance with TEM, the hydrodynamic diameter of AuNP2 suggests that no aggregation occurred during the ligand exchange.

The AuNP surface charge, determined with zeta potential measurements (details in the Methods section) was slightly modified from −13 ± 3 mV for AuNP1 to 0 ± 4 mV for AuNP2 (Table 1) but remained relatively neutral^40^. Neutral nanoparticles often lead to a better biocompatibility, in contrast to positively charged ones, because of the latter’s potential interactions with DNA and some components of the cell membrane^41,42^. Since mucins are negatively charged, one of the strategies to improve mucoadhesion is to use positively charged molecules. However, this could lead to side effects^5^. Regarding negatively charged nanoparticles, they are often less toxic but could lead to electrostatic repulsion with mucins. The neutral charges observed for AuNP1 and AuNP2 made them appropriate for the main objective of this study.

Considering an average core diameter of 7 nm for both AuNP1 and AuNP2, and with the elemental analysis values for Au and S content, the number of internal thiol groups was estimated to be 2689 for AuNP1 and AuNP2 (Appendix 1). According to the elemental analysis, 1205 PEG 2000 of AuNP1, corresponding to 45% of the surface ligands, were replaced by PEG double thiol in AuNP2 (Table 1 and Appendix 1). Combining the TEM images with the results of elemental analysis (32.1% Au, 1.33% S for AuNP1 and 30.5% Au, 1.83% S for AuNP2), the molecular weights of the AuNP were determined as detailed in Appendix 1 (Table 1)^38^. The molecular weights of AuNP1 and AuNP2 were respectively 7 460 000 and 6 255 000 g.mol^−1^. The maximum of absorbance intensity was reported at the wavelength of 517 nm for AuNP1 and 523 nm for AuNP2 at different concentrations ranging from 0 to 0.5 mg.mL^−1^ (Supplementary Fig. S2). The maximal concentration corresponds to 67 nM for AuNP1 and 80 nM for AuNP2. Linear relations can be observed for both types of AuNP until an absorbance of 1.85 for AuNP1 and 1.25 for AuNP2. From these data, their molar extinction coefficients were calculated according to the Lambert-Beer law by measuring the absorbance of AuNP at different concentrations (Supplementary Fig. S2). The molar extinction coefficient is 2.78 × 10^7^ L.mol^−1^.cm^−1^ for AuNP1 and 1.57 × 10^7^ L.mol^−1^.cm^−1^ for AuNP2.

After the synthesis and characterization of AuNP1 and AuNP2, the interactions of these two types of AuNP with mucins were investigated by PAS coloration, UV-visible and fluorescence spectroscopies.

### Mucoadhesive properties of gold nanoparticles

#### Quantitative study of adsorbed mucins

Several techniques to study mucoadhesion have been developed to quantify and characterize the ability of different materials to interact with mucins^1,43–46^. A new protocol used to quantify adsorbed mucins was adapted from different sources in order to study the mucoadhesion of AuNP (details in the Methods section). The periodic acid/Schiff’s reagent (PAS) coloration was initially proposed by Mantle and Allen^47^ in 1978 and used to analyze the mucins content of different samples and the mucoadhesive properties of materials^48–50^. Periodate first oxidizes vicinal hydroxyl groups of the saccharides to aldehydes^50^. In parallel, basic fuchsin, initially pink-purple colored, is decolored by the addition of sodium metabisulfite; the central carbon of the triarylmethane system becomes saturated and its electrons are no longer highly delocalised^51^. Upon mixing the oxidized mucins and the decolored fuchsin, the compounds react and the fuchsin regains a highly delocalized π system.

The challenge associated with probing the binding of mucins to AuNP with this method comes from the red color appearing in the process which is usually analyzed at 555 nm. However, AuNP have also a high absorbance at this wavelength because of their plasmon band. It is thus crucial that all the AuNP are removed from the solution before proceeding to the coloration of the samples, because if even a small amount is left it can influence the UV-visible spectra once the coloration reagents are added. Moreover, the plasmon band of AuNP is greatly affected by the presence of both reagents (Supplementary Fig. S3) and these modifications would interfere with mucoadhesion analysis. As a result, the first step was to determine, by UV-visible spectroscopy, the experimental conditions of centrifugation required to allow the complete precipitation of the AuNP. Different conditions were tested to determine the ideal conditions and the following conditions were selected. Two centrifugations were performed at 4 °C (details in the Methods section): first 30 minutes at 128 000 *g* and then 15 minutes at 356 000 *g*. The first condition should lead to a soft precipitation of mucins binding AuNP without applying a strong force risking their separation, whereas the second centrifugation condition allows the precipitation of AuNP that remained soluble. The mucins alone remained soluble during the process.

After the PAS coloration, the maximum absorbance of the sample resulting from mucoadhesion was compared to the mucins calibration curve. While the maximum absorbance considered in the literature is at 555 nm, full UV-visible spectra after the coloration procedure revealed that the maximum observed was at 575 nm (Supplementary Fig. S4). The absorbance at this wavelength was thus considered for the PAS coloration experiments since it better reflects the evolution of the color over time.

**Figure 2:**
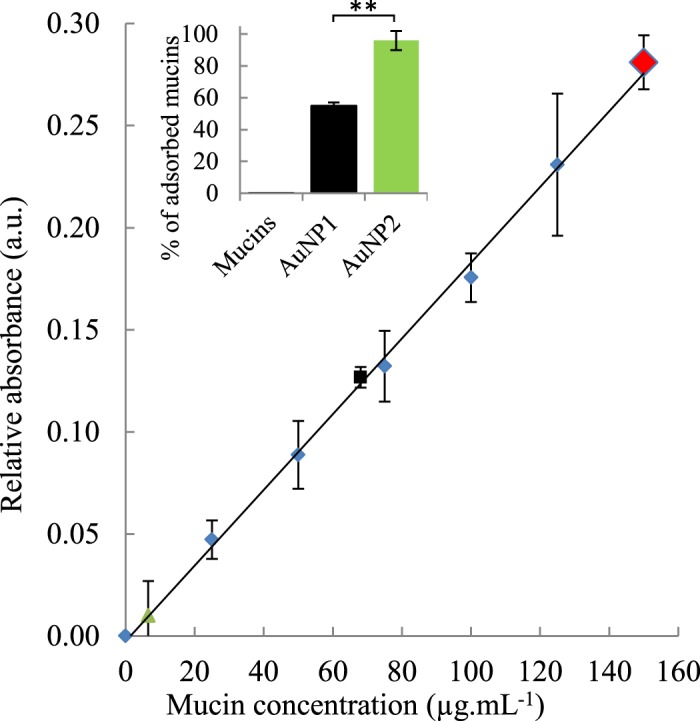
Quantitative analysis of mucins adsorbed on gold nanoparticles. The calibration curve of mucins is represented by blue diamonds. The black square and the green triangle represent the percentage of mucins still free after the mix between, respectively, AuNP1 and AuNP2 (1 mg.mL^−1^) with an initial mucins concentration of 150 µg.mL^−1^ (red diamond). Insert: Histogram of the percentage of adsorbed mucins on AuNP1 and AuNP2 and the control of mucins alone. Data are reported as mean ± standard deviation, n = 3 (P-value < 0.01).

In order to quantify the percentage of mucins interacting with AuNP by PAS coloration, the reagents, the mucins standards and the samples were independently prepared before performing the coloration and the analysis (details in the Methods section). A concentration of 150 µg.mL^−1^ of mucins led to an initial absorbance of 0.28 ± 0.01 at 575 nm, and is represented by the red diamond on the Fig. 2. After performing the PAS coloration protocol to quantify the free mucins, an absorbance of 0.13 ± 0.01 and 0.01 ± 0.02 was respectively observed for mucins previously incubated with 0.1 mg.mL^−1^ of AuNP1 (black square) and AuNP2 (green triangle). These values mean that 55 ± 2% and 96 ± 6% of mucins were adsorbed on AuNP1 and AuNP2 respectively (Insert of Fig. 2). The same experiment was performed with 0.84 mg of AuNP2, corresponding to the same molar concentration of AuNP1, and 90 ± 2% of mucins were adsorbed on AuNP2. Our two types of AuNP thus demonstrate excellent mucoadhesive properties. Moreover, AuNP2 seem to interact with more mucins than AuNP1. As a comparison, Lian *et al*. were able to remove about 220 out of 500 µg of mucins (44%) with 2 mg of nanomicelles in a comparable PAS coloration experiment^48^. Dhawan *et al*. used a much larger amount of microspheres (20 mg) to bind, at best, around 150 out of 500 µg of mucins (30%)^49^. It is noteworthy that in our case, the high-speed centrifugation required to pellet the AuNP might affect the efficacy of the PAS coloration protocol. Indeed, the soft centrifuge conditions used for the PAS coloration are still quite harsh on the bonds between the AuNP and the mucins because the AuNP are much smaller and stable than the nanomicelles or chitosan microspheres aforementioned^48,49^. However, these new conditions still allow an efficient quantitative analysis of the mucoadhesion of very stable particles, which are sought in nanomedicine.

AuNP can exhibit strong mucoadhesive properties because their binding to the mucins could occur mainly via two specific assets of AuNP: (1) by the formation of S-S bonds with the thiol groups of AuNP at the core or at the periphery, or eventually (2) by direct interaction of the cysteine groups with the gold core through the formation of Au-S bonds. The presence of peripheral thiol groups on AuNP2 could explain the difference observed with AuNP1. Indeed, in the case of AuNP1 where mucins interact near the core, steric hindrance of PEG chains could prevent the access of a large number of proteins. Moreover, the Au-S bond possibly formed between mucins and AuNP is slightly weaker than an S-S bond. Their dissociation energies are respectively 254 and 277 kJ.mol^−1^ for Au-S and S-S bonds^52^. The Au-S bond between AuNP and mucins could thus be less resistant to high centrifugations speeds, leading to a partial release of the mucins in the soluble supernatant. However, it is not surprising that AuNP2 strongly interacts with mucins, since thiolating polymers usually result in an improved mucoadhesive potential for a given particle^53^. The mucoadhesion was then characterized by UV-visible spectroscopy in order to potentially identify the formation of bonds near the metallic core through the perturbation of AuNP plasmon band.

#### Localization of the interaction between mucins and gold nanoparticles

The plasmon band of the AuNP is very sensitive to their environment, and modifications of intensity and wavelength of this band indicate changes around the metallic core. UV-visible spectroscopy was thus used to assess the potential of mucins to disturb the metallic core, pointing out an interaction between the protein and the core.

**Figure 3:**
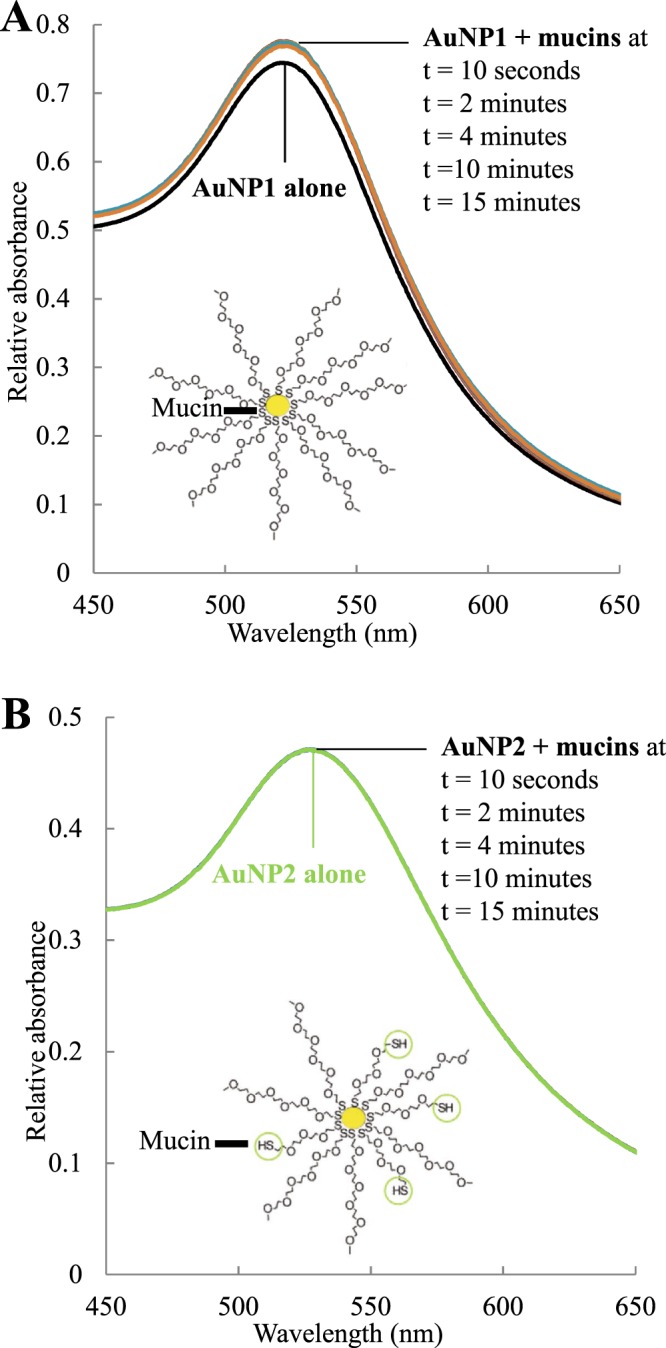
UV-visible spectra of AuNP1 (**A**) and AuNP2 (**B**) alone (0.2 mg.mL^−1^) and in the presence of mucins (0.3 mg.mL^−1^) at different times from 10 seconds to 15 minutes. The suggested interaction between mucins and gold nanoparticles is illustrated in each case.

After incubation of mucins (0.3 mg.mL^−1^) with AuNP (0.2 mg.mL^−1^) for 2 h at 37 °C, the mucoadhesion was assessed by UV-visible spectroscopy. The presence of mucins greatly affects the plasmon band of AuNP1 (Fig. 3A), while the spectrum of AuNP2 remains the same (Fig. 3B). The maximum intensity of the AuNP1 plasmon band increases from 0.74 to 0.77. Because mucins show no absorption in that range of wavelengths, it indicates that the chemical environment around the gold core is modified. This intensity increase suggests a strong perturbation of the gold core, and thus an interaction between mucins and the gold core. As illustrated in Fig. 3A, mucins could form bonds between the thiol groups of their cysteines and the gold core, explaining the important change of the plasmon band of AuNP1. However, the spectrum of AuNP2 remains similar both in the presence and absence of mucins. These data suggest that mucins will only interact with the nanoparticle periphery. The thiol groups of cysteines could form disulfide bonds with the peripheral thiol groups of AuNP2, as illustrated in Fig. 3B, without perturbing the metallic core. The difference of localization could explain the results observed with the PAS coloration. Indeed, the periphery of AuNP is more reachable and a larger number of mucins can interact with it. The experiment was performed after 10 seconds and 2, 4, 10 and 15 minutes (Fig. 3). Interestingly, the absorbance increase observed with AuNP1 instantaneously occurs and remains stable over the time, suggesting that mucins quickly interact with AuNP. This property is quite encouraging for their use as drug delivery systems. Indeed, AuNP could quickly interact with mucosa, increasing the drugs retention times, their bioavailability and thus their efficacy. If mucins deeply interact with AuNP1, their fluorescence properties should be modified. Fluorescence spectroscopy was thus used to confirm AuNP mucoadhesion by characterizing the intrinsic fluorescence changes of mucins.

#### Fluorescence spectroscopy measurements for mucoadhesion characterization

**Figure 4:**
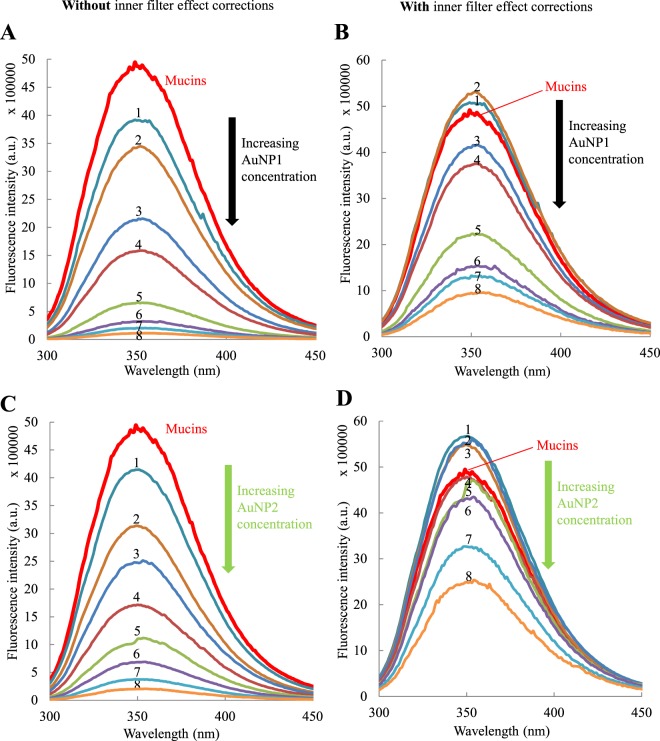
Quenching of mucins fluorescence (0.1 mg.mL^−1^) in the presence of different concentrations of AuNP1 without (**A**) or with (**B**) the application of the global correction factor for the inner filter effect with (equation (1)), and AuNP2 without (**C**) or with (**D**) the application of the global correction factor for the inner filter effect with (equation (1)). The concentrations of AuNP2 are (1) 0.05, (2) 0.1, (3) 0.15, (4) 0.2, (5) 0.3, (6) 0.4, (7) 0.5 and (8) 0.6 mg.mL^−1^. The excitation wavelength is 285 nm.

Fluorescence spectroscopy was used to gather information on the mucoadhesive properties of AuNP. Fluorescence spectroscopy allows the detection of interactions between fluorescent molecules, like mucins, with a second type of molecules in solution, AuNP in our case. This technique measures fluorescence emission spectra according to different exciting wavelengths. Fluorescence emission is proportional to the fluorescent molecule concentration within a certain range of concentrations^54^. Quenching of fluorescence, i.e., a decrease in the fluorescence intensity, can be observed if the fluorescent species interact with a suitable molecule, leading to deactivation. This technique previously provided information on the interaction between components of the mucosal layer and mucoadhesive molecules^6,55^. Indeed, mucins, which are the main components of the mucosal layer, contain different fluorescent amino acids. An excitation wavelength at 285 nm excites both tyrosine and tryptophan residues, but the emission is dominated by tryptophan^54^. The emission spectrum of mucins alone is represented in Fig. 4 (thick red line). In the presence of different concentrations of AuNP1 (Fig. 4A) and AuNP2 (Fig. 4C), the fluorescence intensity of mucins decreased upon increasing the concentration of AuNP. A decrease of 16%, 36%, 50%, 65%, 77%, 86%, 92% and 96% was observed in the presence of 0.05, 0.1, 0.15, 0.2, 0.3, 0.4, 0.5 and 0.6 mg.mL^−1^ of AuNP1. Similarly, a decrease of 16%, 36%, 50%, 65%, 78%, 86%, 92% and 96% was observed in the presence of the same concentrations of AuNP2. However, it is important to note that AuNP absorb light at both the emission and excitation wavelengths and that these data have to be corrected before analysis. Moreover, the two types of AuNP do not absorb the same amount of light at the same concentration because they do not have the same molar extinction coefficient (Supplementary Fig. S2 and Table 1). The correction factor applied to the raw data, taking into account these absorbances, and the corrected data are described in the following section.

#### Inner filter effect and correction factor

**Figure 5:**
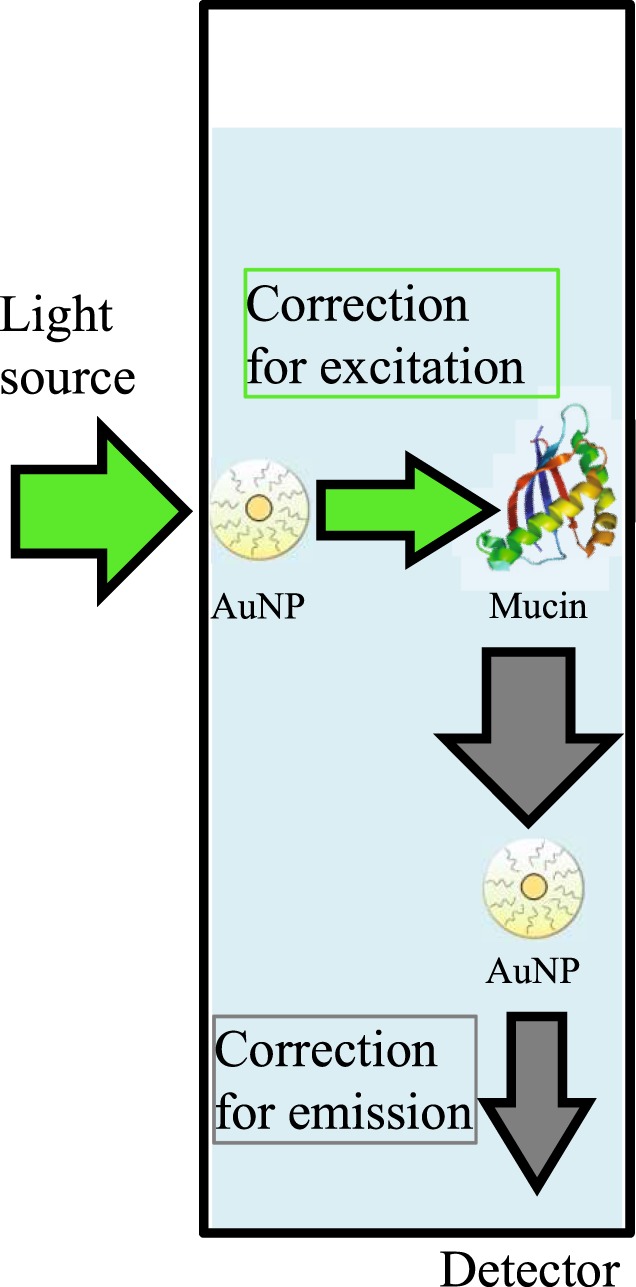
The two corrections taken into account in the global correction factor for the inner filter effect.

As AuNP absorb the incident light, the light intensity reaching the mucins decreases. This phenomenon, called inner filter effect, results in a smaller fluorescence emission and skews the observed results^56^. A correction must be applied to raw data to remove this effect and only detect the quenching due to the interaction between the different species. The correction should also take into account the correction for emission, as illustrated in Fig. 5. In our case, AuNP absorb at the excitation wavelength, decreasing the incident light beam reaching mucins, and also absorbs at the emission wavelength of the mucins, decreasing the detected fluorescence intensity.

Several correction factors have been developed over the years to remove the inner filter effect, considering various parameters such as absorbance values at excitation and/or emission wavelengths or cuvette dimensions^54,57–62^. Special correction factors, often very complex, have also been developed for specific situations like highly absorbing or diffusing media^63–68^, mirror-containing cuvettes^69^, or using external detector as an optical probe^70^, or liposomes^71^. Another correction factor has also been established with a setup requiring a mobile cuvette to measure the fluorescence at two different points. However, without this particular setup, this correction is difficult to use^59^.

**Figure 6:**
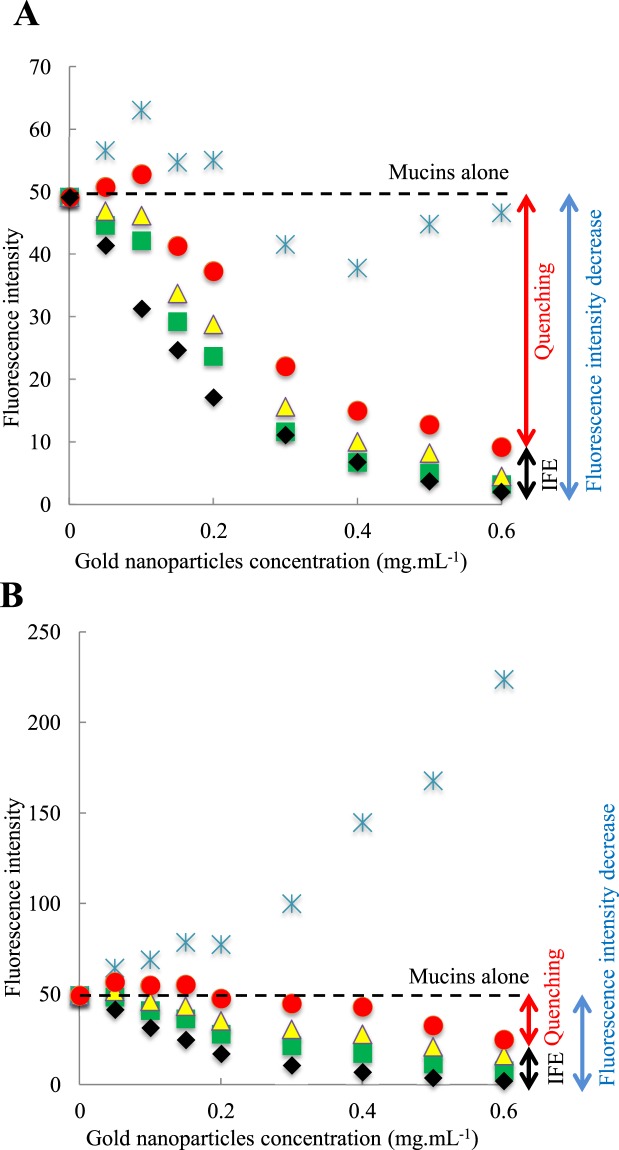
Comparison of mucins fluorescence intensity (0.1 mg.mL^−1^) corrected with the different equations in the presence of several concentrations of AuNP1 (**A**) and AuNP2 (**B**). The raw data are symbolized by black diamonds, the data corrected with correction factor 1 by yellow triangles, with correction factor 2 by blue stars, with correction factor 3 by green squares and with the global correction factor (equation (1)) by red circles. IFE: Inner Filter Effect.

Very few correction factors consider the absorbance values at emission wavelengths, while this correction cannot be discarded when molecules absorbs the emission light, as illustrated in Fig. 5. Two corrections factors commonly used in the literature, here referred to as correction factors 1 (Supplementary equation (S6))^58^ and 2 (Supplementary equation (S7))^54^, take into account this correction at emission, but the obtained data (Supplementary Figs S5 and S6) are not consistent in our case (see Supplementary equations in Appendix 2, and discussion below). We also applied correction factor 3 (Supplementary equation (S5))^57,62^, which is another correction factor largely used in the literature. However, it only considers correction for the excitation and is here shown just for the sake of comparison (see equations in Appendix 2 and Supplementary Fig. S7). The correction factor 1 (yellow triangles) led to similar results as those obtained with correction factor 3 (green squares), as detailed in Fig. 6A for AuNP1 and 6B for AuNP2. The correction factor 2 (blue stars) does not change significantly the results for AuNP1 and leads to aberrant results for AuNP2 as the corrected value of intensity fluorescence is larger than the initial mucins fluorescence, represented as the dotted black line (Fig. 6 and Supplementary S6).

Despite the numerous correction factors found in the literature, none can be consistently applied to our data, considering that AuNP absorb light at excitation and emission wavelengths. We propose a global correction factor (equation (1)), taking properly into account these two effects. The first part of the equation^60,72^ addresses the excitation correction, and the second part, the emission correction (re-absorption of the fluorescence light)^73^. The latter effect is usually negligible in the case where thin cuvettes are used, but is critical in our work where the strong absorption of the AuNP cannot be neglected:

1 $$\frac{{F}_{corr}}{{F}_{obs}}=\frac{{A}_{extot}}{{A}_{exfluo}}(\frac{1-{10}^{-{A}_{exfluo}}}{1-{10}^{-{A}_{extot}}})\times \frac{2.303\,{A}_{emtot}}{(1-{10}^{-{A}_{emtot}})}$$

where A_ex fluo_, A_ex tot_ and A_em tot_ respectively represent the absorbance of the fluorophore at the given excitation wavelength, absorbance of the total solution at the given excitation wavelength and absorbance of the total solution at the given emission wavelength. While other correction factors weakly correct raw data, except for one which leads to aberrant results (Fig. 6B), our equation allows access to corrected data considering the inner filter effect at both excitation and emission (Fig. 4B,D). This precaution prevents overestimating our results, as is often the case in the literature. Upon AuNP interaction, our equation is the one that gives rise to the strongest correction (Fig. 6A,B), as it was anticipated, with the exception of the correction factor 2, which leads to an unrealistic increase of protein fluorescence. The contributions of the inner filter effect (IFE, black arrow) and the quenching (red arrow) compared to the total decrease of the fluorescence intensity (blue arrow) are illustrated in Fig. 6 for the largest AuNP concentration. For all AuNP concentrations, the difference observed between the black diamonds (raw data before correction) and the red circles (corrected data with the global correction factor) corresponds to the inner filter effect. Similarly, the difference observed between the red circles and the dotted line (initial fluorescence intensity of mucins alone) corresponds to the quenching. The very slight over-correction observed for the global correction factor at low concentrations, observed for the values above the initial fluorescence intensity of mucins represented by the dotted black line, could be due to the uncertainty of the method. This new global factor, considering correction at both excitation and emission wavelengths, could become a reference in the field of fluorescence spectroscopy.

**Figure 7:**
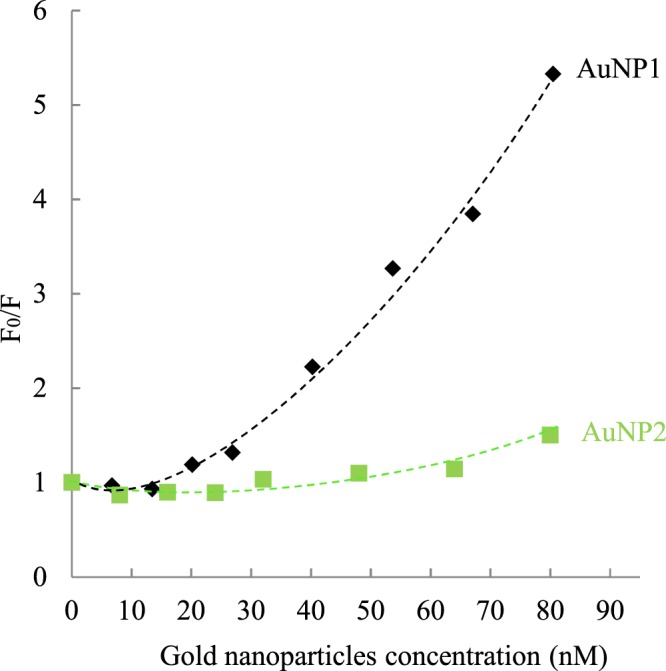
Stern-Volmer plots for mucin fluorescence (0.1 mg.mL^−1^) in the presence of different concentrations of AuNP1 (black diamonds) and AuNP2 (green squares). The dotted lines are a guide to the eye.

From the corrected data with the global correction factor, the quenching effect was then described with a Stern-Volmer plot (Fig. 7)^6,74^. The plot for both AuNP1 and AuNP2 leads to upward curvatures. These non-linear plots could illustrate both collisional and static quenching, meaning that AuNP can dynamically interact with mucins and that some sustainable complexes may also be formed. However, it should be stressed that a FRET mechanism where tryptophan is the donor and AuNP the acceptor is also a very likely process^75^, due to the strong spectral overlap of tryptophan emission and AuNP absorption. In this latter case, a Stern-Volmer formalism could not be applied. The intrinsically complex topology of the system, which should consider at least distance distribution functions, prevents derivation of an exact model, at least from steady-data. In this case, we will not analyze the Stern-Volmer plot in order to obtain an equilibrium constant for the protein-particle interaction. Nevertheless, the plot (Fig. 7) reveals that AuNP1 have a greater photophysical interaction with fluorescent amino acids than AuNP2. Since tryptophan and tyrosine are hydrophobic, mucins must be able to reach the core of AuNP1 more deeply (either for quenching or FRET), leading to a stronger mucoadhesion. This observation supports the results previously observed with PAS coloration and UV-visible spectroscopy. Indeed, the thiol groups present in the periphery of AuNP2 will interact firstly with the mucins, preventing a deep interaction with the thiol groups near the core, and thus lead to a weaker quenching of mucin fluorescence. In comparison, AuNP1 only present thiol groups near their core, leading to a deeper interaction and thus to a stronger quenching of mucins fluorescence.

One can note that control nanoparticles for AuNP can be difficult to establish. Indeed, the specific arrangement of AuNP makes them unique and thus hardly comparable. The two types of AuNP proposed here allow us to study the influence of the presence of thiol groups in periphery. However, it is also interesting to determine the role of the PEG in the mucoadhesion phenomenon. Indeed, two controversial roles of this polymer in mucoadhesion are described in the literature^76^. On the one hand, PEG chains can get entangled with the sugar-rich domains of the mucins, which is a strategy already known to explain the mucoadhesive properties of polymeric nanoparticles^77^. However, low molecular weight PEG is not mucoadhesive when coupled at high density with polystyrene nanoparticles^77,78^. For comparison purposes, the mucoadhesion of polymeric nanoparticles similar to our AuNP (details in the Methods section) were thus studied. These nanoparticles were mainly composed of PEG in periphery and contain poly(lactic-co-glycolic acid) as their core instead of gold. Their hydrodynamic diameter, 51 ± 2 nm, was determined by DLS (details in the Methods section) and was in the same order of magnitude. Their mucoadhesive properties could only be assessed by fluorescence spectroscopy because the absence of a plasmon band (due to the lack of a gold core) and their instability against the centrifugations conditions prevent the feasibility of respectively UV-visible spectroscopy or PAS coloration experiments. Moreover, as these nanoparticles do not absorb light at either the excitation or the emission wavelengths, the inner filter effect does not need to be considered for the fluorescence measurements. The experiments showed that no quenching of mucins fluorescence was observed in the presence of different concentrations of polymeric nanoparticles ranging from 0.04 to 0.43 mg.mL^−1^ (Supplementary Fig. S8). These data strongly suggest that it was not the PEG in itself that was involved in the mucoadhesion observed for AuNP. In these conditions, the presence of the gold core and/or thiol groups appears to be crucial for the interaction of AuNP with mucins.

## Conclusion

Two types of AuNP were synthesized and characterized. AuNP1 contains only thiol groups near the metallic core whereas AuNP2 contains thiol groups at the core and at the periphery. They both have similar properties regarding their core diameters, hydrodynamic diameters and charges. Their mucoadhesive properties were investigated using three different techniques that involved a significant optimization of the protocols due to the specific properties of AuNP. The PAS coloration allowed the quantification of the amount of mucins adsorbed on AuNP with a largely adapted protocol considering new speed of centrifugations and readings at different wavelengths. AuNP2 interact with a larger number of mucins at its periphery. The UV-visible spectroscopy measurements support the fact that mucins interact with the core of AuNP1 whereas they remain at the periphery of AuNP2. Finally, the mucoadhesion study by fluorescence spectroscopy allowed the highlighting of the interaction of mucins and AuNP by the quenching of the mucins fluorescence. A new global correction factor for the inner filter effect, considering the correction at both excitation and emission wavelengths, was described and used for analysis. The fluorescence quenching of mucins is larger in the presence of AuNP1, allowing to conclude that these AuNP1 could interact strongly with internal amino acids of mucins. Moreover, no fluorescence quenching of mucins is observed with polymeric nanoparticles without a gold core and thiolated ligands, suggesting that the Au-S and/or S-S bonds are responsible for the mucoadhesion of the AuNP. The different techniques used to study the AuNP strongly highlighted their mucoadhesive properties, which can be modulated through the use of peripheral thiol groups. Eventually, since it is known that AuNP have good drug encapsulation properties, it should be possible to produce AuNP for drug delivery that are highly mucoadhesive and that would improve the medication thanks to their longer residence time on their mucous target.

## Methods

### Material

Gold chloride trihydrate (HAuCl_4_∙3H_2_O), sodium borohydride (NaBH_4_), chlorhydric acid (HCl), nitric acid (HNO_3_), sodium chloride (NaCl), basic fuchsine (pararosaniline hydrochloride) and sodium metabisulfite were purchased from VWR International. Polyethylene glycol dithiol with a molecular weight of 1000 g.mol^−1^ (referred to as PEG double thiol) and periodic acid were purchased from Sigma-Aldrich (St. Louis, MO). Polyethylene glycol methyl ether thiol with a molecular weight of 2000 g.mol^−1^ (referred to as PEG 2000) was purchased from Laysan Bio (Arab, AL). Dichloromethane and methanol were purchased from Fisher Scientific, and mucins from bovine submaxillary gland were purchased from Cedarlane Laboratories (Burlington, ON). All the glassware used for the synthesis of AuNP was first thoroughly washed with *aqua regia* (3:1 HCl:HNO_3_) and rinsed with nanopure water.

### Synthesis of gold nanoparticles AuNP1

AuNP coated with PEG 2000 (referred to as AuNP1) were synthesized using a single phase Brust method, modified from a technique previously published^27^. A significant advantage of this procedure is the absence of tetraoctylammonium bromide (TOAB), thus avoiding residual ionic contamination of this toxic compound, important for an eventual application in ophthalmology. First, a stock solution of aqueous gold chloride was prepared by dissolving 1 g of HAuCl_4_∙3H_2_O to a final concentration of 0.1 mg.mL^−1^. Then, 0.5 mL (0.127 mmol) of this stock solution was added to 30 mL of a 1:1 methanol:water solution. 0.048 g (0.024 mmol) of PEG 2000 and 20 mL of methanol were then added, and the solution was agitated for 90 minutes. 0.040 g (1.057 mmol) of NaBH_4_ dissolved in 20 mL of ice-cold water was then added dropwise at a rate of 1 mL.min^−1^ under vigorous stirring using a peristaltic pump. The solution was stirred for 3 h to ensure proper growth and stabilization of the particles^79^. The methanol was then evaporated under reduced pressure with a rotary evaporator, and the nanoparticles were extracted with dichloromethane using a minimal amount of a saturated NaCl solution. AuNP1 were then dissolved in 10 mL of water and dialysed (molecular weight cut-off of 12000 g.mol^−1^) over two days. The nanoparticles were recovered and kept in solution in water in amber glass bottles. The final concentration of the solution was determined by weighing the freeze dried nanoparticles in a given volume.

### Synthesis of gold nanoparticles AuNP2: ligand exchange

Eight molar equivalents of PEG double thiol, with respect to the amount of PEG 2000 used for the initial synthesis, were dissolved in the AuNP1 solution. The mixture was stirred for 5 days in the dark under argon atmosphere, and then purified by extensive dialysis (molecular weight cut-off of 12000 g.mol^−1^) over 4 days. The resulting nanoparticles, referred to as AuNP2, were recovered and kept in solution in amber glass bottles.

### UV-visible spectroscopy

UV-visible spectra were recorded using the Cary 50 Bio UV-vis, from Varian. For quantitative mucoadhesion experiments, disposable plastic cuvettes (10 × 10 mm pathlength) from Brand (#759200) were used. For localization experiments as well as for absorbance data for inner filter effect correction, Hellma (#111.057-QS) fluorescence quartz cuvettes (5 × 5 mm pathlength) were used. All the experiments for the characterization of AuNP1 and AuNP2, as well as for the mucoadhesion characterization, were performed twice and the measurements were highly reproducible.

### Transmission electron microscopy

Copper grids covered with a vaporized carbon film were purchased from Ted Pella (California, USA). AuNP were deposited on the grid and left for one minute before removing the excess volume with a filter paper. The image acquisition was performed with a JEOL JEM 1230 microscope (Tokyo, Japan). The zoom factor was 50 000x. More than one hundred AuNP were counted and analyzed with ImageJ to determine the number and the diameter of the gold cores.

### Dynamic light scattering and Zeta Potential

Dynamic light scattering (DLS) and Zeta potential measurements were performed with the NanoBrook Omni from Brookhaven Instruments Corporation. The solutions were analyzed in 10 mM KNO_3_ at 25 °C at an angle of 90° using the CONTIN mode for DLS, and phosphate-buffered saline (PBS) for Zeta potential. After an equilibrium time of ten minutes, ten measurements of 120 seconds were performed for each sample and the values were reported as the Effective Diameter. Two different concentrations of AuNP (6 nM and 16 nM for AuNP1 and 15 nM and 23 nM for AuNP2) were analyzed for both techniques and lead to the same results. Disposable plastic cuvettes from Eppendorf (#952010051) and Brookhaven (BI-SCP), as well as an electrode for aqueous solutions (BI-ZEL) were used for DLS and zeta potential measurements.

### Elemental analysis

The samples were sent to the Saskatchewan Research Council (SRC) Geoanalytical Laboratories. Sulfur and gold content of the AuNP were determined by induction furnace and fire assay, respectively. Briefly, sulfur analysis was performed with an SC-144DR C/S induction furnace, manufactured by LECO. The samples were first heated to a very high temperature under pure oxygen. During combustion, sulfur was released into SO_2_ and measured with infrared cells. The gold content was determined by fire assay. The samples were weighed and heated before the extraction of the precious metals which were analyzed by inductively coupled plasma atomic emission spectroscopy (ICP-AES).

### Preparation of the mucins solution

Typically, a stock solution of mucins from bovine submaxillary glands was prepared by dissolving the crude powder in water to a final concentration of 0.40 mg.mL^−1^. The solution was sonicated for 30 minutes and the pH was adjusted to 7.4. It was then kept at 4 °C if not used immediately. A sonication of 30 minutes was performed before each new dilution.

### Fluorescence spectroscopy

Fluorescence spectra of mucins and mucoadhesion samples were obtained with a Fluorolog-3 (Horiba). Fluorescence spectra for mucoadhesion of polymeric nanoparticles (see the section *Synthesis of polymeric nanoparticles*) were obtained with a Cary Eclipse fluorescence spectrophotometer from Varian. A 5 × 5 mm light path quartz cuvette from Hellma Analytics was used (#111.057-QS). Excitation wavelength showed optimal results at 285 nm and the scans for emission were thus recorded from 300 to 450 nm. All the experiments were performed twice and the measurements were highly reproducible.

### Periodic Acid/Schiff’s Reagent coloration

The methodology of the Periodic Acid/Schiff’s Reagent (PAS) coloration is based on previously published protocols^48–50^. However, several parameters were optimized because of the specific properties of AuNP. Data are reported as mean ± standard deviation, n = 3. Parametric two-tailed independent t-test analysis were used to compare means between experimental groups and a P-value of < 0.01 was considered statistically highly significant.

#### Determination of the centrifugation conditions

Before proceeding the PAS coloration, the slowest speed and minimal time required to pellet all the AuNP were determined on an OPTIMA-MAX (130 000 rpm) ultracentrifuge from Beckman Coulter. Optimal conditions were 30 minutes of centrifugation at 128 000 *g* followed by 15 minutes at 356 000 *g*, at 4 °C. In these conditions, the mucins remained soluble after centrifugation in the absence of AuNP.

#### Preparation of the reagents for the PAS coloration

Periodic acid reagent: Fresh reagent was prepared by mixing a 1:700 ratio of aqueous periodic acid 50% m/V (4 °C, protected from light) with aqueous acetic acid 7% V/V, and covered with Parafilm before each coloration.

Schiff’s reagent: A fuchsin stock solution was prepared by adding 20 mL of 1 M HCl to 100 mL of an aqueous 1% basic fuchsin solution. Before coloration, 50 mg of sodium metabisulfite was added to 3 mL of the stock solution, carefully covered with several layers of Parafilm and incubated for 2 hours at 37 °C. The reagent was cooled to room temperature for a few minutes before use.

#### Preparation of samples

Standards of mucins were prepared to a final concentration of 0, 25, 50, 75, 100, 125 and 150 µg.mL^−1^ to obtain a calibration curve. Samples used for mucoadhesion studies were prepared in triplicate incubating mucins (150 µg.mL^−1^) with different concentrations of AuNP. Control solutions containing only AuNP were also prepared. Standards, samples and controls were incubated under stirring for 2 hours, and centrifuged as previously described.

#### Coloration protocol

A volume of 180 µL of periodic acid reagent was added to 600 µL of the supernatant. After 2 hours of incubation at 37 °C, 60 µL of the Schiff’s reagent was added and reacted for precisely 30 minutes before UV-visible spectra measurements (400–800 nm).

#### Analysis

Each control solution was subtracted from the corresponding mucoadhesion sample. A calibration curve was drawn using the maxima of the mucins standards peaks at 575 nm and used for quantitative analysis.

### Synthesis of polymeric nanoparticles

Poly(lactic-co-glycolic acid)-PEG 5000 g.mol^−1^ polymeric nanoparticles were synthesized by dropwise addition of 1 mL of the polymer solution in acetonitrile to 10 mL of ultrapure water under magnetic stirring at 1400 rpm^80^. To obtain 50 nm nanoparticles, the solution contained 5.5 mg.mL^−1^ of Poly(lactic-co-glycolic acid) 10000 g.mol^−1^-PEG 5000 g.mol^−1^ and 4.5 mg.mL^−1^ of Poly(lactic-co-glycolic acid) 28000 g.mol^−1^-PEG 5000 g.mol^−1^. The nanoparticles were purified by filtration on 100 kDa Amicon centrifugal filters.

## Electronic supplementary material

Supplementary information
