# Supplementary material for: Insights into gold nanoparticles as a mucoadhesive system
Source: Sci Rep. 2018 Sep 25;8:14357. doi: 10.1038/s41598-018-32699-2 (PMC6156509; doi:10.1038/s41598-018-32699-2)
Supplement: Supplementary file 1 — Supplementary information [file 41598_2018_32699_MOESM1_ESM.pdf]

Insights into gold nanoparticles as a mucoadhesive system

Mathieu Ouellette, Florence Masse, Mathilde Lefebvre-Demers, Quentin Maestracci, Philippe Grenier, Robert Millar, Nicolas Bertrand, Manuel Prieto, Élodie Boisselier*

Supplementary Information

		Page
Figure S1	TEM images and size histograms of the metallic core of AuNP	S2
Figure S2	Determination of molar extinction coefficients	S3
Figure S3	UV-visible spectra of AuNP with PAS coloration reagents	S4
Figure S4	UV-visible spectra of mucins after PAS coloration	S5
Figure S5	Correction with correction factor 1 (equation (S6))	S6
Figure S6	Correction with correction factor 2 (equation (S7))	S7
Figure S7	Correction with correction factor 3 (equation (S5))	S8
Figure S8	Fluorescence quenching with polymeric nanoparticles	S9
Appendix 1.	Equations for the calculation of AuNP estimated molecular weights	S10
Appendix 2.	Equations of the different corrections factors	S11

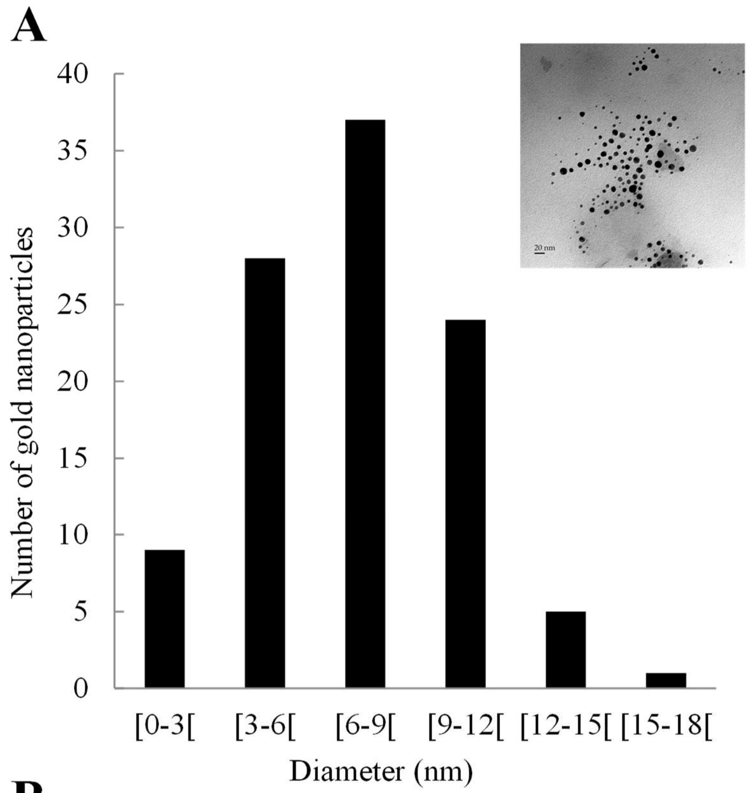

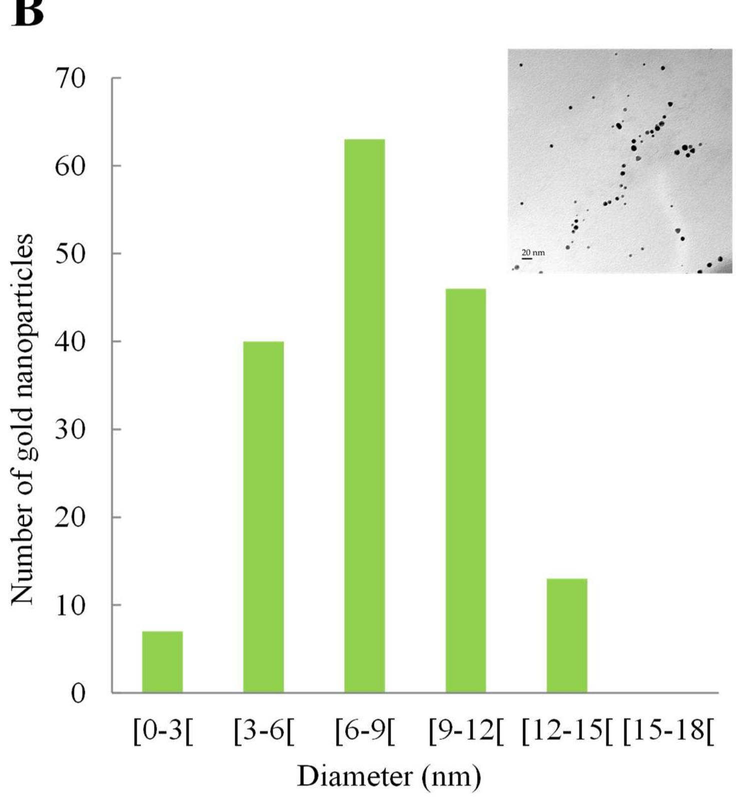

Figure S1. Size histograms of the metallic core of AuNP1 (**A**) and AuNP2 (**B**). Inserts: TEM images of AuNP1 (**A**) and AuNP2 (**B**) with a scale bar of 20 nm.

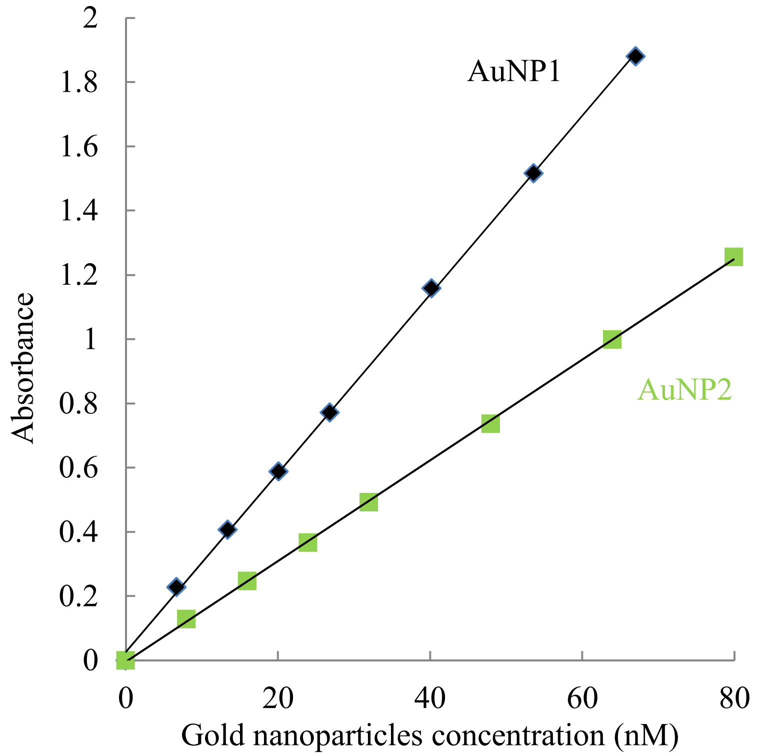

Figure S2. Absorbance values for different concentrations of AuNP1 (7, 13, 20, 27, 40, 54 and 67 nM) at 517 nm (black diamonds) and AuNP2 (8, 16, 24, 32, 48, 64, 80 nM) at 523 nm (green squares). The molar extinction coefficient is 2.78 x 10^7 $L.mol^{-1}.cm^{-1}$ for AuNP1 and 1.57 x 10^7 $L.mol^{-1}.cm^{-1}$ for AuNP2.

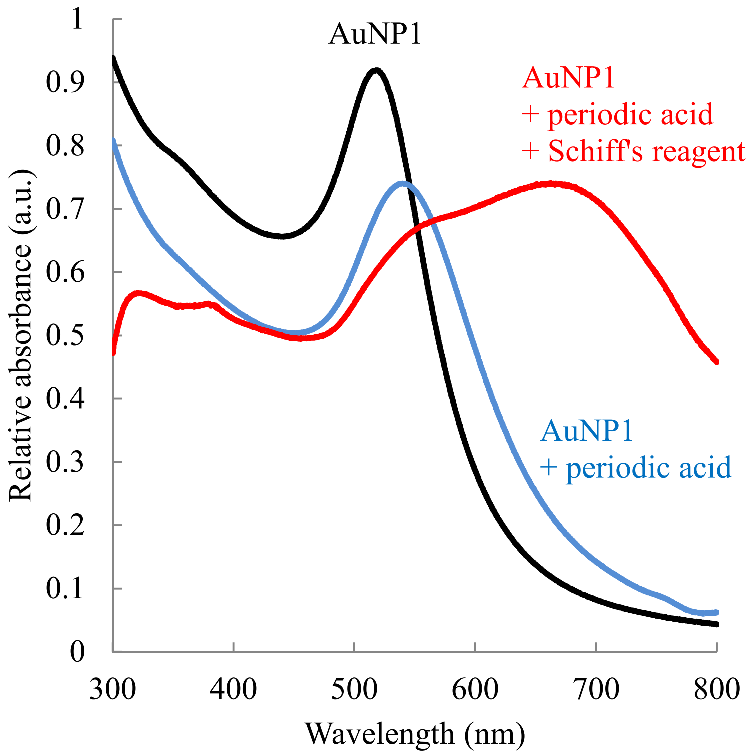

Figure S3. UV-visible spectra of AuNP1 alone (in black) and in the presence of PAS coloration reagents: AuNP1 with periodic acid (in blue) and AuNP1 with periodic acid and Schiff's reagent (in red). The reagents spectra were also performed as blank for the different conditions of mucoadhesion samples and subtracted from the corresponding spectra.

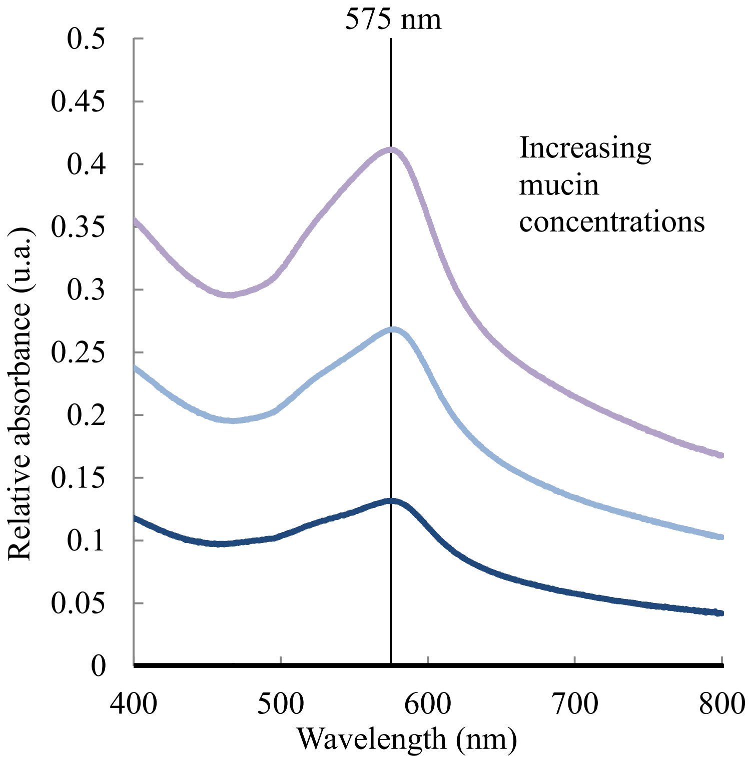

Figure S4. UV-visible spectra of mucins at different concentrations (50, 100 and 150 $\mu g.mL^{-1}$) after PAS coloration. The maximal absorbances are observed at 575 nm.

A

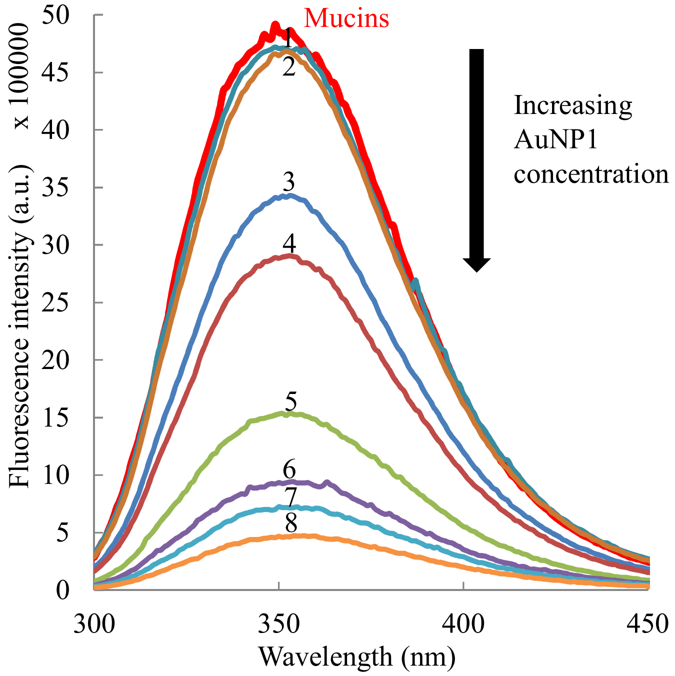

B

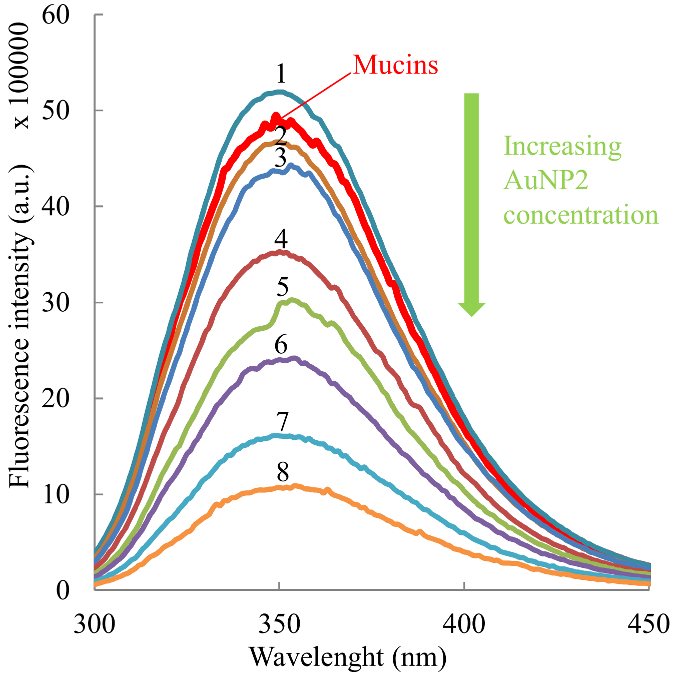

Figure S5. Quenching of mucins fluorescence (0.1 mg.mL^{-1}) in the presence of different concentrations of AuNP1 (**A**) and AuNP2 (**B**) with correction factor 1 (Gauthier's equation, equation (S6)). The concentrations of AuNP are (1) 0.05, (2) 0.1, (3) 0.15, (4) 0.2, (5) 0.3, (6) 0.4, (7) 0.5 and (8) 0.6 mg.mL^{-1}. The excitation wavelength is 285 nm.

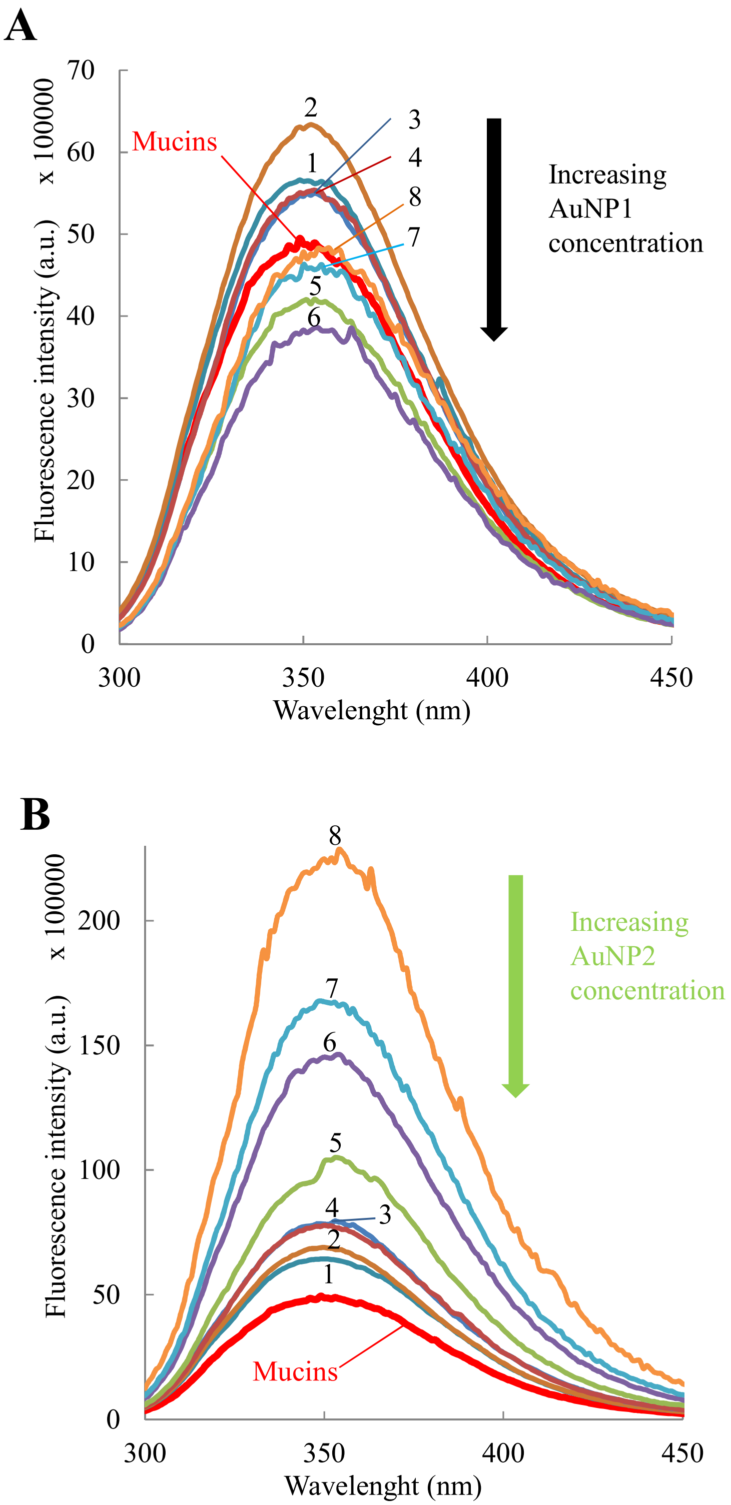

Figure S6. Quenching of mucins fluorescence (0.1 mg.mL^{-1}) in the presence of different concentrations of AuNP1 (**A**) and AuNP2 (**B**) with correction factor 2 (Lakowicz's equation, equation (S7)). The concentrations of AuNP are (1) 0.05, (2) 0.1, (3) 0.15, (4) 0.2, (5) 0.3, (6) 0.4, (7) 0.5 and (8) 0.6 mg.mL^{-1}. The excitation wavelength is 285 nm.

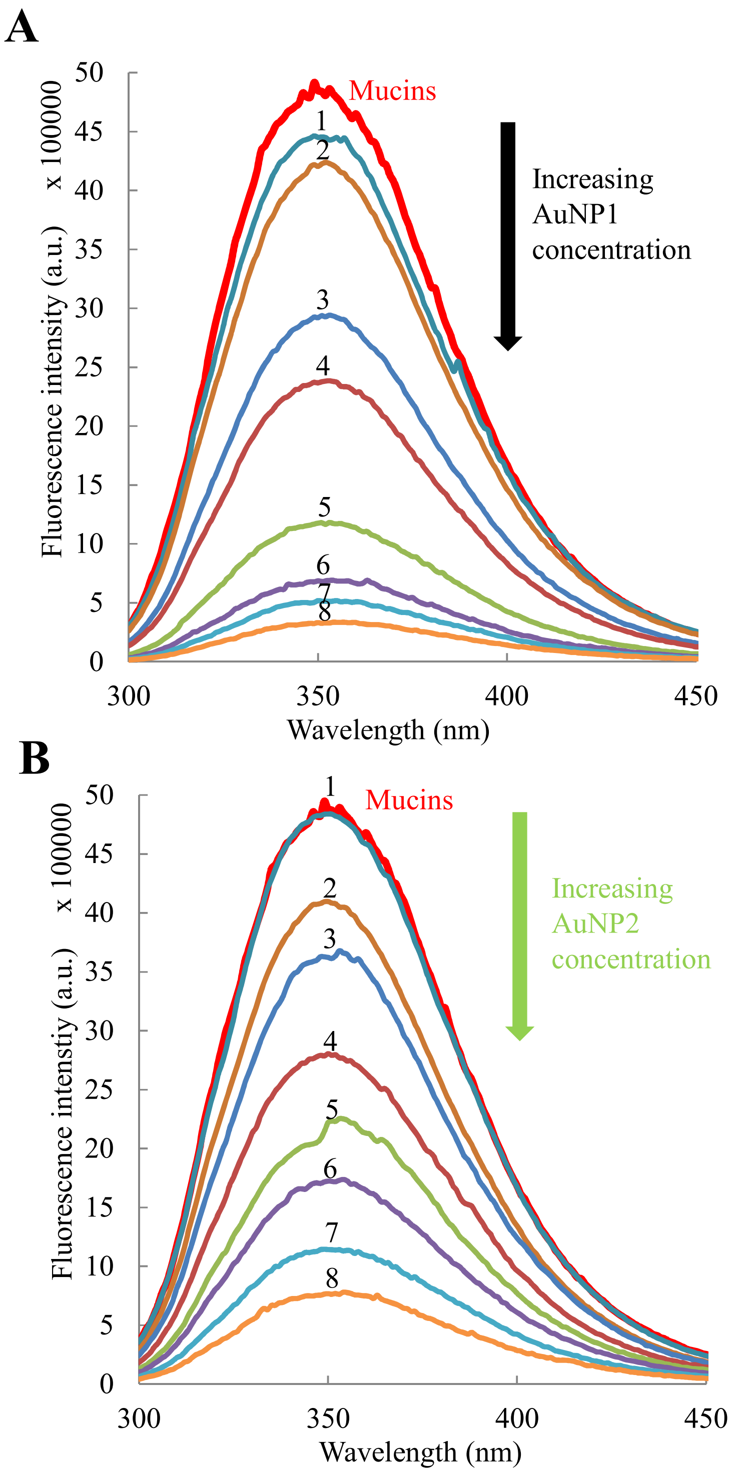

Figure S7. Quenching of mucins fluorescence (0.1 mg.mL^{-1}) in the presence of different concentrations of AuNP1 (**A**) and AuNP2 (**B**) with correction factor 3 (Parker's equation, equation (S5)). The concentrations of AuNP are (1) 0.05, (2) 0.1, (3) 0.15, (4) 0.2, (5) 0.3, (6) 0.4, (7) 0.5 and (8) 0.6 mg.mL^{-1}. The excitation wavelength is 285 nm.

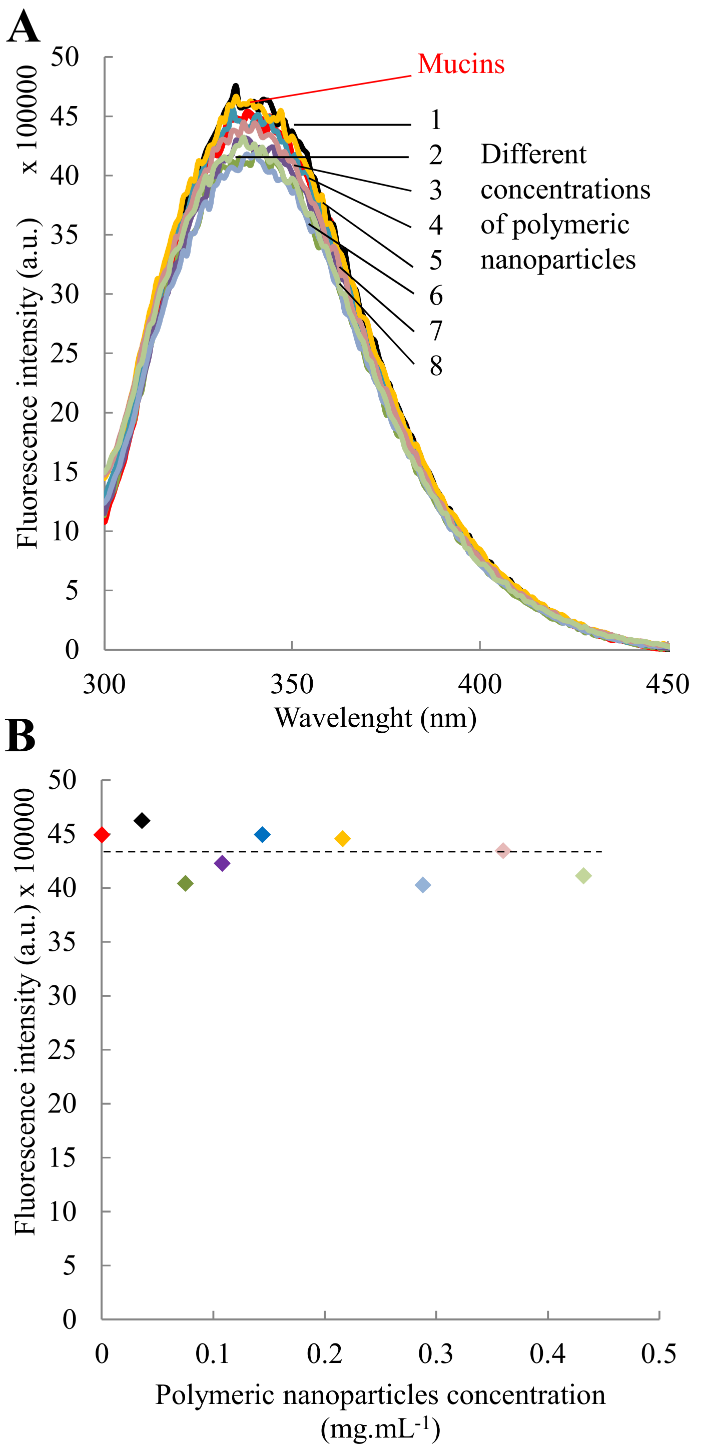

Figure S8. Quenching of mucins fluorescence (0.1 mg.mL^{-1}) in the presence of different concentrations of polymeric nanoparticles (**A**). The concentrations are (1) 0.04, (2) 0.08, (3) 0.11, (4) 0.14, (5) 0.22, (6) 0.29, (7) 0.36 and (8) 0.43 mg.mL^{-1}. The excitation wavelength is 285 nm. The fluorescence intensity of mucins at 344 nm is represented in respect to the concentration of polymeric nanoparticles (**B**).

Appendix 1. Equations for the calculation of AuNP estimated molecular weights

The molecular weights of the gold nanoparticles were estimated as previously published (Boisselier et al, Chem. Comm. 2008, 5788-5790). Assuming monodisperse spherical gold nanoparticles, the volume (V) of the metallic core can be estimated according to:

$$V = \frac{4\pi \times (\text{radius in Å})^3}{3} \qquad \textbf{(S1)}$$

The number of gold atoms present in the gold core, n (Au), can be estimated according to:

$$\text{n (Au)} = \frac{\text{V in Å}^3}{17\ \text{Å}^3} \qquad \textbf{(S2)}$$

With the molecular weight (MW) of Au and S, the elemental analysis allows the estimation of the number of thiol groups, n (S), on the surface according to:

$$\frac{\text{n (Au)} \times \text{MW (Au)}}{\text{\% Au atoms}} = \frac{\text{n (S)} \times \text{MW (S)}}{\text{\% S atoms}} \qquad \textbf{(S3)}$$

Finally, the molecular weight of AuNP can be estimated according to:

$$\text{MW (AuNP)} = (\text{n (Au)} \times \text{MW (Au)}) + (\text{n (S)} \times \text{MW (thiolated ligand)}) \qquad \textbf{(S4)}$$

Appendix 2. Equations of the different corrections factors

Equation parameters were homogenized for more clarity.

2.1 Parker's equation – Correction factor 3

Historically, one of the first correction factor used to correct the inner filter effect was derived by Parker et al. in 1957[1] and published in the book *Photoluminescence of solutions* in 1969:[2]

$$\frac{F_{corr}}{F_{obs}} = \frac{2.303A(x_2 - x_1)}{10^{-Ax_1} - 10^{-Ax_2}} \quad \textbf{(S5),}$$

where x_1 and x_2 are the cuvette dimensions between the cuvette edge, and respectively the frontal and the distal extremity of its aperture. The published data deal with the influence of benzoin concentration on the formation of the fluorescent borate-benzoin compound. This equation is the starting point of all the other correction factors adapting their equation according to their specific conditions. Limitations of this equation are its application only for solutions with an absorbance lower than 2.0, and the fact that the absorbance at the emission wavelength is not considered.

2.2. Gauthier's equation – Correction factor 1

In 1986, Gauthier et al. optimized Parker's equation with the consideration of the width of the cuvette (d), the excitation beam width (s), the distance between the excitation beam and the cuvette edge (g) and the absorbance at both the excitation (A_{ex}) and the emission (A_{em}) wavelengths as follows:[3]

$$\frac{F_{corr}}{F_{obs}} = \frac{2{,}3d\,A_{ex}}{1-10^{-dA_{ex}}} \times 10^{gA_{em}} \times \frac{2{,}3s\,A_{em}}{1-10^{-sA_{em}}} \quad \textbf{(S6)}$$

In 1991, Pulchalski et al. compared equations (S5) and (S6) for a study of quinine sulphate and concluded that equation (S6) was more accurate because the absorbance at the emission wavelength was taken into account.[4]

2.3 Lakowicz's equation – Correction factor 2

In 1983, Lakowicz et al. developed a correction factor also considering the absorbance at both the excitation (A_{em}) and the emission (A_{em}) wavelengths as follows:[5]

$$\frac{F_{corr}}{F_{obs}} = 10^{[\frac{A_{ex}+A_{em}}{2}]} \quad \textbf{(S7)}$$

Pulchaski et al. also compared this equation with equations (S5) and (S6) and concluded that the results were in accordance with the ones obtained with the other equations, although less linear.[4]

All these equations are easy to use and do not require any special equipment, unlike the one referenced in Lutz et al.[6] According to the specific conditions of the studied quenching, some of them are more appropriate due to the consideration of absorbance at excitation and at emission wavelengths. However, the global correction factor proposed in our manuscript (equation (1)), allows a complete correction of inner filter effect and thus an accurate quenching study without underestimating the re-adsorption of the light by compounds in solution.

2.5 References

1. Parker, C. A.; Barnes, W. J., Some experiments with spectrofluorimeters and filter fluorimeters. *Analyst* **1957,** *82* (978), 606-618.
2. Stegemeyer, H., Photoluminescence of Solutions. *Angew. Chem. Int. Ed.* **1969,** *81* (23), 1007-1008.
3. Gauthier, T. D.; Shane, E. C.; Guerin, W. F.; Seitz, W. R.; Grant, C. L., Fluorescence quenching method for determining equilibrium constants for polycyclic aromatic hydrocarbons binding to dissolved humic materials. *Environmental Science & Technology* **1986,** *20* (11), 1162-1166.
4. Puchalski, M. M.; Morra, M. J.; von Wandruszka, R., Assessment of inner filter effect corrections in fluorimetry. *Fresenius' Journal of Analytical Chemistry* **1991,** *340* (6), 341-344.
5. Lakowicz, J. R., *Principles of Fluorescence Spectroscopy*. 3 ed.; Springer US: 2006.
6. Lutz, H.-P.; Luisi, P. L., Correction for Inner Filter Effects in Fluorescence Spectroscopy. *Helvetica Chimica Acta* **1983,** *66* (7), 1929-1935.
